# High-Intensity Interval Exercise: Methodological Considerations for Behavior Promotion From an Affective Perspective

**DOI:** 10.3389/fpsyg.2021.563785

**Published:** 2021-01-28

**Authors:** Allyson G. Box, Steven J. Petruzzello

**Affiliations:** Department of Kinesiology and Community Health, University of Illinois Urbana-Champaign, Champaign, IL, United States

**Keywords:** HIIT, physical activity, valence, pleasure, HIFT, affect

High-intensity interval exercise and high-intensity interval training (HIIT), an exercise approach alternating short bouts of vigorous exercise with less intense recovery or rest periods, has been rated as a leading fitness trend, ranked between #1 and #3 in the annual survey of “Top 20 Worldwide Fitness Trends” since 2014, with no sign of weakening “popularity” (Thompson, 2019). This increased popularity in HIIT programs has been mirrored by a subsequent uptick in related research. In such investigations, HIIT appears to deliver important physiological benefits (much like those observed by any regular exercise behavior; Kilpatrick et al., 2014; Jelleyman et al., 2015). However, little is known about why high-intensity interval programs have gained such popularity within the fitness industry (i.e., increasing number of high-intensity interval type franchises such as F45^®^ Training, Orangetheory Fitness^®^, Crossfit^®^ Training, Bootcamps, and so on), let alone, and perhaps most importantly, whether such a regimen encourages prolonged exercise^1^ behavior (i.e., adherence). Based on previous work (e.g., Williams et al., 2008) with continuous exercise, the affective experience of such HIIT-type programs may be a particularly important reason why it is popular. Thus, we will address some methodological concerns pertaining to HIIT research, specifically related to the study of affective states. We will also propose potential solutions for investigating such psychological phenomena associated with this popular exercise regimen.

## Disentangling HIIT Terminology

HIIT, by definition, utilizes planned intensity and work to rest/recovery ratios in unlimited variations (see Laursen and Buchheit, 2019, Figure 1 for detail on intensity and design variability). That is, a ratio defined as 1:1 (keeping in mind that numerous ratios can be delineated) may differ in duration (e.g., 30-s work: 30-s rest/recovery; 2-min work: 2-min rest/recovery) and exercise type (e.g., running; cycling; also consider that body weight and resistance circuits often include multiple movements within a single HIIT session). These types of protocols are almost exclusively done in research. A closely related regimen, referred to as HIFT, is defined as a “training style that incorporates a variety of functional movements, performed at high-intensity (relative to an individual's ability), to improve parameters of general physical fitness and performance” (Feito et al., 2018, p. 2).

**Figure 1:**
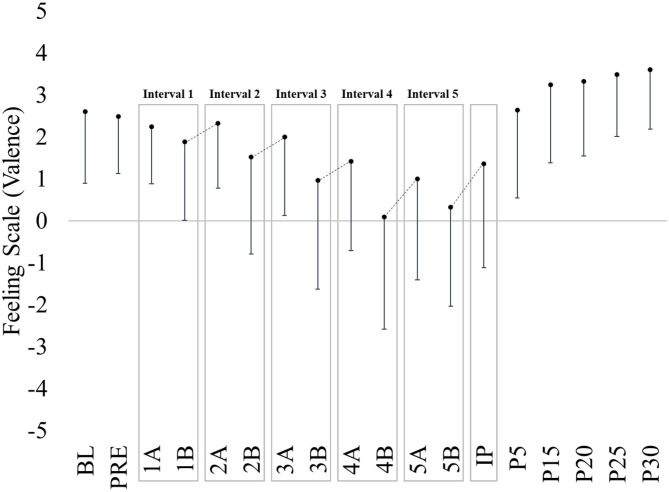
Evidence of affective rebound phenomena during interval exercise. The present figure has been adapted from data and figures presented within Box et al. (2020). Regular exercisers (*n* = 25) performed an imposed high-intensity interval exercise on a cycle ergometer (5, 3-min exercise intervals interspersed with 1-min rest) resulting in 75–95% of HR_peak_ throughout the exercise session. Affective valence was recorded within the first (A) and last (B) 10-s of each exercise interval. See Box et al. (2020) for additional experimental design detail. BL, baseline; PRE, immediately prior to exercise while sitting on cycle ergometer; IP, immediately post exercise while remaining on cycle ergometer; P, post exercise. Affective rebounds are delineated by a dashed line.

HIFT is distinct from HIIT in at least two important ways: (a) rest/recovery and (b) intensity. While rest/recovery in research-based HIIT is planned and synchronous, HIFT provides greater autonomy in both rest and recovery. That is, the individual makes the determination of when, and for how long, to rest/recover. Another consideration is whether “high-intensity” is an adequate term to express the intensity performed. Due to the flexibility in high-intensity interval programming, laboratory studies *impose* a range of intensities from just within “vigorous” intensity all the way to “supra-maximal” (Laursen and Buchheit, 2019). However, the fitness communities (e.g., F45^®^ Training, Orangetheory Fitness^®^, Crossfit^®^ Training, Bootcamps, and so on) applying “high-intensity interval-type” programming encourage the individual to “exercise as hard as you can,” which may or may not equate to a physiological index of high intensity (see American College of Sports Medicine et al., 2018, p. 146). It is possible the majority of individuals^2^ engaging in leisure- or health-related “interval-type” exercise are most often engaging in ***self-selected, perceived high-intensity (i.e., completely autonomous) interval-type*** exercise. Thus, intensity itself is perceptual in nature and varies during this type of exercise session. As a result, the individual is likely modifying their work-to-recovery ratio, and subsequently their intensity, based on how they are feeling (i.e., their affective experience). It is entirely possible these “interval-type” programs remain a popular form of exercise due to the autonomy in both level of exercise-intensity and rest/recovery, resulting in an affective quality (e.g., less unpleasant) that enhances affective associations toward exercise.

## Maximizing Quality of Research on Affective Responses

Psychological Hedonism is, stated simply, the idea that human behavior is a result of an innate pursuit of pleasure and avoidance of displeasure (Young, 1952; Rozin, 1999). Indeed, accumulating evidence suggests in-task (i.e., during exercise) affective states (i.e., pleasure vs. displeasure) are associated with an increased likelihood of continued exercise behavior, even up to 6- and 12-months later (Williams et al., 2008; see also Rhodes and Kates, 2015 for a review). While we will not go as far to say hedonics is the only, or even primary, concept to be considered in human behavior, we do implore that ***affective states*** (also considered feeling states^3^) should at least be considered. As Antonio Damasio (1994) eloquently wrote -

“*Knowing about the relevance of feelings in the process of reason does not suggest that reason is less important than feelings, that it should take a backseat to them or that it should be less cultivated. On the contrary, taking stock of the pervasive role of feelings may give us a chance of enhancing their positive effects and reducing their potential harm (p. 246)*.”

With so much yet to learn about feeling states in the context of exercise behavior, the relationship between exercise intensity and affective valence (i.e., pleasure vs. displeasure) has been well-established. Evidence has repeatedly demonstrated the ventilatory threshold (VT) as the biological marker of most importance for influencing ***affective states***, that is, fluctuations in one's affective state reliably occur depending on the exercise intensity relative to the VT. Intensities below the VT elicit very little fluctuation in affective state and result in primarily homogenous in-task pleasure, while exercise intensities above the VT elicit intense fluctuation in affective state and result in homogenous in-task displeasure. More fascinating is the effect of exercise intensity at, or proximal to, the VT. It is within this intensity range where heterogeneity of affective responses exists, with such heterogeneity being attributed to fitness, personality, and other individual differences (Acevedo et al., 2003; Ekkekakis et al., 2005; Box and Petruzzello, 2020).

While yet to be empirically tested, it is likely individuals who regularly engage in (self-selected, perceived) high-intensity interval type exercise (i.e., not in laboratory-based studies) are choosing to perform at an intensity at or proximal to their VT when prompted to “exercise as hard as you can.” Again, it is likely the autonomy in exercise-intensity and rest/recovery (adjusting both exercise-intensity and rest/recovery when it feels necessary) allows for more effective affective rebounds. Further, these affective rebounds, occurring immediately following exercise cessation within each of the rest/recovery intervals and at the end of the overall exercise session (see Figure 1, revised from Box et al., 2020 to demonstrate interval-exercise affective rebounds), likely result in a different affective response than if intensity or rest/recovery were imposed at pre-planned intervals. Thus, experiencing decreases in pleasure (or increases in displeasure) within successive imposed high-intensity work intervals likely is experienced very differently than when the work-to-recovery intervals are self-selected (i.e., autonomous). Differences in affective responses have been observed with a slight preference toward self-selected vs. imposed continuous exercise (Oliveira et al., 2015), but this has yet to be empirically demonstrated during interval-type exercise.

## Considerations for Experimental Design

High-intensity interval exercise is not, nor will any exercise mode ever be, the only solution for increasing exercise behavior. This is not to say that high-intensity exercise research is frivolous; rather quite the opposite. An attempt should be made to best understand how exercise of any variety influences an individual, physiologically ***and*** psychologically, and how the individual chooses and adheres to different modes of activity. We are urging the utmost caution in designing and implementing research to examine such high-intensity, interval type exercise by thoughtfully considering the question at hand, deliberately designing a protocol that unequivocally tests these questions, and transparently interpreting the findings so as not to obscure the answers within.

We strongly encourage the following considerations when examining high-intensity interval-type exercise with an eye toward exercise promotion. We believe the inclusion and transparency of these variables will aid in comparing and interpreting findings across the “interval-type” literature.

We suggest that investigators:

a) ***Appropriately acquire and report a physiological index of intensity (e.g.*,** ***%HR***_***max***_**,** ***%VO***_**2*peak***_***)*** ***and/or total accumulated work***. Given the demonstrated importance of intensity on affective responses, this is a crucial methodological step. The decision of which physiological intensity index is dependent on the research question and experimental limitations, but whenever possible there should not be a sole reliance on perceived exertion.
b) ***Attempt to acquire and quantify VT alongside in-task affect***. The importance of this marker stems from the well-established evidence that affective responses are most aligned with the VT rather than a percentage of maximum heart rate or VO_2peak_. This is important because at the same physiological index of intensity (e.g., %VO_2peak_), one person could be well above their VT and experience the exercise as unpleasant while another person is well under their VT and experiences the exercise as pleasant. Thus, it is more informative to have a participant's in-task affect displayed and interpreted in relation to their VT. This also allows for easier comparisons across studies implementing various interval designs and exercise movements.
c) ***Record affective states during both work and rest/recovery periods along with pre- and post-exercise***. The time-of-assessment should be identical between any compared conditions (especially interval vs. continuous exercise), meticulously designing the assessments to occur *during* exercise for both conditions. The timing should be such that affective states are acquired at least immediately prior to the exercise session (within 1-min), during exercise ***and*** rest/recovery intervals, and immediately post (within 1-min) exercise session. At minimum, affective states should be assessed within the first and last 15-s of each exercise interval and halfway through rest/recovery. Waiting to assess affect until the work bout has just ended will not capture the same affective dynamic as the affective rebound is likely already taking place at that time. Participant reactivity to repeated assessments is also possible when acquiring affective states several times within a short time period, thus forethought is needed to determine how often it is necessary to assess affective states. See Ekkekakis (2013) for suggestions on affective state questionnaires.
d) ***Manipulate only one exercise variable (i.e., intensity, duration, or mode) while standardizing all other variables between testing conditions***. It is necessary, until evidence has been established, to manipulate only one exercise variable to determine whether affective states are a consequence of intensity, duration, or mode. Too often in the high-intensity interval-type vs. moderate-intensity continuous exercise literature all three variables are manipulated, resulting in muddled interpretations. This will likely require multiple experiments in order to confidently provide inferences to a single question, but careful planning that allows for both replication and extension of findings should provide more confidence in the findings.
e) *P****reemptively control, recognize, record, and report possible extraneous variables***. Affective states, by definition, fluctuate moment-to-moment and many variables could unduly influence an individual's affective state data. Consider the lab environment (e.g., decorative pictures, number of research staff, music, unintended conversation, etc.), the researcher's appearance and tone (e.g., white coat affect, provocative clothing, excited vs. bored tone, etc.), the participant's personal items (e.g., cell phone, smart watch, etc.), and so on to eliminate as many potentially confounding sources as possible.

## Conclusion

HIIT programs and the numerous variations that have evolved are popular and seem destined to be part of the exercise landscape. Our position in this paper is that we, as exercise behavior researchers, need to exert much greater care in the way that we study these high-intensity interval options. We have outlined what we think are the most critical issues in the design and execution of research, particularly as related to understanding the affective dynamics of such exercise. Given that affect has been shown to be intimately linked with exercise intensity and that affect experienced during exercise is consistently predictive of adherence, careful examination of these affective dynamics in high-intensity interval exercise is crucial. The bottom line is that, as popular as some forms of activity might be, if people do not experience them in such a way that engenders long-term adherence, which is likely if the activity is experienced as unpleasant, it really does not matter what physiological benefits might be gained. We have a lot of work to do to achieve this level of understanding, particularly with high-intensity interval exercise.

## Author Contributions

AB was responsible for initial drafting, while both AB and SP equally contributed to the content and draft finalizing. Both authors contributed to the article and approved the submitted version.

## Conflict of Interest

The authors declare that the research was conducted in the absence of any commercial or financial relationships that could be construed as a potential conflict of interest.

## Footnotes

^1^While the field often uses the term “exercise” behavior/promotion, this should be interpreted as physical activity behavior and promotion, not simply exercise. The goal is to increase the total amount of physical activity, which may be accumulated via any bodily movement. Exercise, by definition, is planned, structured, repetitive physical activity that is often performed to improve one or more aspects of health (e.g., physical, mental, and social) and fitness (e.g., cardiorespiratory endurance, muscular strength and endurance, flexibility; American College of Sports Medicine et al., 2018).

^2^Athletes with a goal-directive of performance are likely using technology, providing a physiological index, to assure they are adequately exercising within a high-intensity range.

^3^Feeling states are more commonly referred to as affective states within the literature and consist of three similar, but distinct, components: core affect, emotion, and mood. Core affect is the most basic, consciously accessible feeling state and is considered to reflect both affective valence (i.e., pleasure vs. displeasure) and activation (i.e., reflecting the psychological state of wakefulness). Emotion is an “intense” fluctuation in core affect following an internal or external event, real or imagined, that dissipates in a relatively quick (i.e., seconds) timeframe. Moods are longer in duration (i.e., hours or days), less intense (i.e., degree of fluctuation in core affect), and are distinguished by their onset ambiguity (see Ekkekakis, 2013, Nowlis and Nowlis, 1956, and Russell, 2003 for further detail on affective states).
